# From petri dishes to politics – a multi-pronged approach is essential for saving endangered species

**DOI:** 10.1038/s41467-018-04962-7

**Published:** 2018-07-04

**Authors:** Terri L. Roth, William F. Swanson

**Affiliations:** Center for Conservation and Research of Endangered Wildlife (CREW), Cincinnati Zoo & Botanical Garden, 3400 Vine Street, Cincinnati, OH 45220 USA

## Introduction

As many species spiral toward extinction, the spotlight shines bright on scientists and assisted reproductive technologies (ARTs) as the last hope for a few. Although it can be a powerful conservation tool, ART alone is not enough to save species from extinction.

Since the 1970s, the potential role of ART in species conservation has been touted by a small but ever-increasing cohort of reproductive scientists committed to their cause. The rapid advancement of ARTs, embraced and incorporated into the human fertility field and livestock industries, emboldened convictions that the same technologies would figure prominently in endangered species breeding programs. Approximately 40 years later, endangered species’ ARTs are in greater demand as populations shrink, genetic diversity is lost and breeding programs falter. In a few cases, ART now represents the only viable strategy for potentially saving genes from certain imperiled individuals or populations like the Northern white rhino sub-species^1^. However, the development of ARTs for wildlife species has not lived up to early expectations^2^. There are isolated reports of progress employing high-tech procedures like cloning, but in almost all cases, they result in lost pregnancies and/or neonatal mortalities, efficiency is extremely low and overall conservation value questionable^3^. In contrast, a plethora of studies have demonstrated that artificial insemination (AI), one of the simplest ARTs, can produce offspring in nearly 60 different exotic mammals and fertile eggs in over 35 non-domestic birds^4^. Regardless, routine use of ARTs in wildlife management remains rare. To date, AI has proven truly beneficial to the genetic management and breeding programs of just three endangered mammals: the giant panda, black-footed ferret, and Asian elephant, though it is peripherally employed to assist managing a handful of other threatened and endangered species. Given the potential power of ART as a conservation tool, it is worth examining why it is not well-established or embraced by those striving to conserve endangered species, the promise it holds and its inherent limitations.

## The reality of ART in species conservation

Scientific challenges of developing ART for wildlife surpassed expectations^2^. The endangered species spectrum is broad, and reproductive physiology differs substantially between and within taxa, making ART a unique science within each species, and rendering the livestock-based paradigm almost obsolete as a point of reference. Furthermore, in contrast to the livestock industry and human fertility fields, few researchers and labs are focused on any one species, funding is scarce, and by definition, endangered species are rare, so research subjects are scant. Typically, the few animals available for experimental ART are those failing to reproduce naturally, likely representing a sub-fertile population. Additionally, wildlife species have not been subjected to the artificial selection pressures for high fertility under intensive management that were imposed on livestock for generations. Therefore, the bar should be lowered regarding efficacy expectations for ART in endangered species. Finally, scientists inherently thrive on discovery, and after demonstrating the feasibility of a specific ART in a particular species, they frequently pursue new challenges. Referred to as the “one-and-done” phenomenon, this approach does little to establish techniques as useful management tools within the wildlife community, and fosters a disconnection between reproductive sciences and conservation applications.

Aside from scientific hurdles (and in discordance with today’s technology-dominated world), animal staff resistance to embracing ART as a management tool for their charges is not unusual. ART often is considered a specialty field that requires outside experts, incurs significant costs and potentially poses unnecessary risks to individual animals. Indeed, ART research and development comes at a price, and scientists are pressured to raise funds, but once established, techniques can be shared at little expense. Conservation impact expands markedly if ART scientists empower others, but too often technology transfer is not a priority. Additionally, by their nature, published studies frequently are based on data subsets that are not inclusive of all attempts. Therefore, the percentage of animals conceiving and producing viable young following ART trends lower in practice than what is reported in the literature, and the cumulative expense of conducting repetitive procedures can become cost-prohibitive. Finally, with increased concern about animal well-being and the preservation of species-appropriate behaviors, opportunities for animals to interact naturally with counterparts for reproduction are a priority, and substituting ART for natural mating is sometimes equated to animal management failure.

## The value of ART as a conservation tool

Despite these scientific, technical and philosophical challenges, the potential value of ART in endangered species conservation efforts has never been greater. Escalating ethical, political and logistical barriers to acquiring animals from native environments for ex situ management have made the establishment of self-sustaining populations a high priority for the world’s zoo community^5^. However, our capacity to improve sustainability is severely limited by inadequate housing space, restricting population sizes below levels necessary to maintain targeted gene diversity for long-term survival. If sperm and embryo cryopreservation in conjunction with AI and embryo transfer, respectively, were successfully integrated into the management strategies for these wildlife species, the required minimum population sizes would decrease substantially. Furthermore, semen collection and banking from wild males would enable genetic infusion from wild to managed populations without removal of live animals from their natural habitats^6,7^. Similarly, international genetic exchange among genetically distinct regional populations could become more commonplace. The precedent for such strategies already exists for cheetahs (Namibia to U.S.)^6^, sand cats (UAE to U.S.), and elephants (South Africa to Europe)^8^. Additionally, sperm and embryos preserved from individuals that fail to reproduce in their lifetimes could be vehicles for infusing their unrepresented genes into populations long after death. The feasibility of this approach has been demonstrated recently in the Indian rhino^9^, Pallas’ cat^10^ and black-footed ferret^11^. Furthermore, despite best efforts, physical and behavioral obstacles occasionally interfere with natural mating between optimal genetic matches, and ART may be needed as a remedy. Finally, the application of ART should not be restricted to intensively managed populations within zoological parks. Arguably, in this 21st century, all wildlife populations will be managed by humans to some degree, and many reserves contain inbred taxa that would benefit from an infusion of new genes. Although animal translocations among wildlife parks are not uncommon, for some species, post-release mortality can be excessive and integration of introduced individuals into existing communities rare.

## ART alone is not the solution

Despite the potential and proven benefits associated with integrating ART into endangered species management programs, ART alone is not the solution to saving species from extinction. Implying otherwise is scientific overreach and can breed complacency among governmental agencies responsible for protecting wildlife. Recent, commendable progress with rhino ART raises hopes that genes from the functionally extinct Northern white rhino subspecies can be conserved^12^. However, impressive results in a Petri dish don’t easily translate into a herd of healthy offspring. Achieving the latter requires navigating an untrodden path fraught with obstacles, and it remains unlikely that a viable population of Northern white rhinos will be restored. To that end, the two primary approaches being pursued include the generation of offspring using Northern white rhino gametes derived from induced pluripotent stem cells, which to-date has only partially been achieved in the mouse^13^ and remains highly speculative for rhinos, and intra-species hybridization^1,12^. If successful, the latter would create Northern × Southern white rhino subspecies crosses, and several generations of intensive inbreeding among these hybrid individuals would be required to dilute the Southern white rhino genes while concentrating those of the Northern white rhino, a strategy likely resulting in severe inbreeding depression, if mating succeeded at all. A contributing factor to the demise of the Northern white rhino was their failure to breed naturally in managed programs. Perhaps more to the point, no single strategy is adequate to save a species. Just as no endangered species has recovered via ART in the absence of a concurrent, successful natural breeding program, wildlife rangers are ineffective without governmental agencies to enforce the law when poachers are arrested, and those regulatory agencies can only enforce laws drafted and ratified by political leaders.

Too much precious time is lost arguing about how scarce conservation dollars should be spent and which approach is most important for conserving species. The reality is that funds available for high-tech scientific approaches like ART often are not transferable to “boots on the ground” conservation measures, and vice versa. Therefore, competition among strategies for the same funding is less common than many propose. Besides, multiple conservation approaches should be pursued concurrently. For decades, debate has raged over protecting wild populations vs. establishing managed breeding programs when it should not be an either/or ultimatum; both efforts should be undertaken. Let the Sumatran rhino be a lesson to us all. Heatedly contested in the 1980s when first proposed, and harshly criticized when it initially faltered^14^, ex situ Sumatran rhino breeding eventually succeeded^15^. Today, many of the same organizations that condemned early efforts now herald managed breeding as the only strategy that can save this species^16^.

In the dynamic landscape of wildlife conservation, it is impossible to accurately predict which strategies will prove pivotal in a species’ recovery, but most assuredly, survival of many hinge on our ability to function in unity employing varied approaches to achieve a common goal. As Aristotle once said, “the whole is greater than the sum of its parts”. That certainly rings true for endangered species conservation efforts. So, let’s congratulate the ranger who apprehends the poacher, reward the community that restores the forest, support the politicians who pass wildlife preservation legislation and celebrate the scientists achieving breakthroughs that advance ART. Any one of these efforts alone will fall short, but working in concert, they may prevail in saving a few imperiled species and minimizing the loss of biodiversity during the Earth’s sixth great extinction event.
